# Experimental Investigation on Geopolymer Concrete with Various Sustainable Mineral Ashes

**DOI:** 10.3390/ma14247596

**Published:** 2021-12-10

**Authors:** Narayanan Subash, Siva Avudaiappan, Somanathan Adish Kumar, Mugahed Amran, Nikolai Vatin, Roman Fediuk, Radhamanohar Aepuru

**Affiliations:** 1 Department of Civil Engineering, University College of Engineering Nagercoil, Nagercoil 629004, India; Subashnarayanan87@gmail.com; 2 Departamento de Ingeniería en Obras Civiles, Universidad de Santiago de Chile, Av. Ecuador 3659, Estación Central 7800002, Chile; 3 Department of Civil Engineering, University VOC College of Engineering, Thoothukudi 628008, India; adishk2002@yahoo.co.in; 4 Department of Civil Engineering, College of Engineering, Prince Sattam Bin Abdulaziz University, Alkharj 16273, Saudi Arabia; 5 Department of Civil Engineering, Faculty of Engineering and IT, Amran University, Amran 9677, Yemen; 6 Peter the Great St. Petersburg Polytechnic University, 195251 St. Petersburg, Russia; vatin@mail.ru; 7 Polytechnic Institute, Far Eastern Federal University, 690922 Vladivostok, Russia; fedyuk.rs@dvfu.ru; 8 Departamento de Ingeniería Mecánica, Facultad de Ingeniería, Universidad Tecnologica Metropolitana, Santiago 1242, Chile; raepuru@utem.cl

**Keywords:** geopolymer concrete, cement concrete, M-sand, sea sand, quarry dust, copper slag, lime stone powder

## Abstract

The aim of this research was to find the best alternative for river sand in concrete. In both geopolymer concrete (GPC) and cement concrete (CC), the fine aggregates are replaced with various sustainable mineral ashes, and mechanical and durability tests are conducted. Specimens for tests were made of M_40_ grade GPC and CC, with five different soil types as river sand substitute. The materials chosen to replace the river sand are manufactured sand (M-sand), sea sand, copper slag, quarry dust, and limestone sand as 25%, 50%, 75%, and 100%, respectively by weight. GPF50 and CC50 were kept as control mixes for GPC and CC, respectively. The test results of respective concretes are compared with the control mix results. From compressive strength results, M-sand as a fine aggregate had an increase in strength in every replacement level of GPC and CC. Additionally, copper slag is identified with a significant strength reduction in GPC and CC after 25% replacement. Copper slag, quarry dust, and limestone sand in GPC and CC resulted in considerable loss of strength in all replacement levels except for 25% replacement. The cost of GPC and CC is mixed with the selected fine aggregate replacement materials which arrived. Durability and cost analyses are performed for the advisable mixes and control mixes to have a comparison. Durability tests, namely, water absorption and acid tests and water permeability and thermal tests are conducted and discussed. Durability results also indicate a positive signal to mixes with M-sand. The advisable replacement of river sand with each alternative is discussed.

## 1. Introduction

Concrete is a material which stands next to water in global usage. About 60 to 80 percent of the concrete is occupied by fine aggregate and coarse aggregate [1,2,3]. River sand as fine aggregate and crushed granite stone as coarse aggregate are the most commonly used materials in concrete. The construction industry is facing a problem in getting aggregates for concrete production due to its scarcity and for the motive of saving nature [4,5]. The use of waste materials and manufactured aggregates as an alternative for natural aggregates in concrete must be entertained to save nature, prevent pollution, and reduce costs [6,7,8,9,10]. Cement and geopolymer are the two binders used to embed the aggregates. Usage of cement is much higher when compared to geopolymer concrete. Geopolymer materials emerged to minimize the air pollution occurring due to Ordinary Portland cement (OPC) production [5]. By upcycling industrial waste/by-products into a high-value construction material required for infrastructure development, geopolymer concrete helps to protect the environment [11,12,13,14,15,16,17,18]. Inorganic waste particle chemical activity provides an alternative to OPC. In recent decades, Geopolymer Concrete has gained favor as a more environmentally friendly alternative to regular OPC [19] Concerns about the high volumes of carbon dioxide emitted by cement production have led to new suggestions for supplemental cementing ingredients, with the goal of reducing the amount of cement used for concrete formation. Many factors affect the strength and workability of geopolymer concrete [20,21]. Among those factors, selecting the powder content plays an important role. Basically, the aluminosilicates used in geopolymer concrete are such things as class F fly ash, ground granulated blast furnace slag (GGBS), kaolin clay, fayalite slag, silica fume, red mud, rice husk ash, geothermal silica, and bentonite; other aluminosilicate and additional silica or alumina sources that are suitable for one-part alkali-activated materials are largely the same as those for conventional alkali-activated materials. Sodium hydroxide and sodium silicate are the most widely employed activators in one-part geopolymer investigations [22,23]. Utilizing mineral admixtures like fly ash and GGBS in GPC may achieve good strength when compared to metakaolin and silica fume. GGBS-based GPC at room temperature and fly ash-based GPC in hot curing have obtained good results [24,25,26]. The geopolymer mix proportion has been obtained for grades up to 80 N/mm^2^ using fly ash, GGBS, metakaolin, silica fume, and rice husk in earlier studies [27]. The cement concrete mix proportion can be obtained by using IS 10262-2019 [28].

Conservation of river sand is a major issue now. Governments are protecting river sand from use by construction companies who cannot use it as before. To minimize the usage of river sand, many researchers have attempted to use GPC and CC with various soil types as fine aggregates [29]. Studies have shown that the presence of M-sand gives good results in GPC and CC [19]. M-Sand boasts better compressive and split tensile strengths of the concrete with aluminosilicate materials, such as silica fume, metakaolin, and GGBS as cement replacement [30,31,32]. Small strength is identified between sea sand and river sand specimens made of GPC. Even the corrosive rate was decreased for geopolymer-reinforced concrete with sea sand. Untreated sea sand can be utilized in CC to reduce the use of natural river sand [33,34]. The results of various studies have indicated that the CC with crushed limestone has shown improved mechanical and rheological properties [35]. Quarry powder, a solid waste, has been utilised to make UHPC, and the newly created UHPC’s properties have been evaluated [36]. The use of up to 22.5 percent quarry dust as fine aggregate in EPS-foam concrete [37,38] resulted in a 30 percent increase in compressive strength [39,40]. In addition, quarry dust and copper slag are used in current research to replace river sand. Concrete containing 40% copper slag have a higher compressive strength than reference mixtures [41]. As the amount of copper slag in the mix increased up to 30%, the compressive and split tensile strengths also increased [42]. To improve the workability and strength of concrete, copper slag as fine aggregate can be entertained [30]. Past studies concludes the optimum use of copper slag as 30% to 60% as a replacement material for river sand [42]. Water demand decreased by up to 22% by using 100% copper slag as fine aggregate in cement concrete [43]. According to previous studies, using large amounts of quarry dust might reduce fluidity and boost compressive strength [44]. Quarry dust is clearly one of the sustainable replacement materials for river sand, according to a study [45,46]. The findings of this study suggest that quarry waste material can be successfully used in GPC [32]. There have been no major studies carried out comparing all the mentioned sand types in concrete.

However, this research concentrates on finding the most suitable substitute from the waste and natural materials for river sand in concrete. Both cement and geopolymer binders are selected to bind the aggregates. The M_40_ grade of GPC and CC are taken to replace the fine aggregate. Manufactured sand, sea sand, limestone sand, copper slag, and quarry dust are considered as river sand replacement materials in this research. The compressive strength test on fine aggregate-replaced specimens was conducted for 7 and 28 days. Durability tests, namely, the water absorption and acid test, and the water permeability and thermal test were conducted and recorded. Cost analyses for respective mixes give an additional advantage to this research.

## 2. Materials and Methods

Six types of fine aggregates were used in this research to study the influence of each material on the properties of concrete. GGBS and fly ash were used as powders in GPC. To obtain GPC, alkaline liquids, namely, sodium silicate and sodium hydroxide were used. The 10M NaOH was obtained by diluting pellets (Astra Chemicals Pvt. Ltd., Chennai, India) in water. Glass silicate was obtained with a water content of 55% and 45% solid content (Na_2_O and SiO_2_). Ordinary Portland cement of grade 53 (Ramco Cement, Chennai, India) was used to get a cement binder. Quarry dust, M-sand, sea sand, limestone sand, copper slag, and river sand (Blue Metals Pvt. Ltd., Chennai, India) were used as fine aggregate. M-sand and quarry dust were obtained from a nearby crusher. The manufacturing of M-sand involves crushing, screening, and washing. Quarry dust is the leftover material from the extraction and processing of rocks to make M-sand [47,48]. The mineral admixtures required were purchased from an admixture supplier of South India (Astra Chemicals Pvt. Ltd., Chennai, India), and the sand types were taken from South Indian land areas. Physical and chemical properties of the mineral admixtures were provided by the supplier Astra Chemicals Pvt. Ltd. Physical properties of fine and coarse aggregates were found as per IS 2386-3 (1963) and IS 383 (1970). The physical and chemical properties of the materials used are given in Table 1, Table 2 and Table 3.

## 3. Mix Proportion Details for GPC and CC

The present investigation was conducted using M_40_ grade GPC and CC concrete. Previous studies prescribed the mix proportions of M40 GPC using GGBS and fly ash [27]. M40 grade CC was designed using IS10262-2019 [28]. In both binders, river sand was replaced by M-sand, sea sand, limestone sand, copper slag, and quarry dust by weights of 25%, 50%, 75%, and 100%, respectively. The designed material quantities according to replacement levels are shown in Table 4 and Table 5. Successively, the specimens are casted as per the mix shown in Table 4 and Table 5. Mix GPF was kept as the control mix for GPC. River sand in mix GPF was fully replaced by quarry dust, copper slag, sea sand, M-sand, and limestone sand. Thus, mixes GPFM1 to GPFL4 arrived. Similarly, CC was kept as the CC control mix. River sand in mix CC was fully replaced by quarry dust, copper slag, sea sand, M-sand, and limestone sand. Thus, we obtained mixes CCFM1 to CCFL4.

## 4. Tests for GPC and CC with Various Fine Aggregates and Coarse Aggregates

Slump cone, compressive strength, split tensile strength, water absorption, water permeability, thermos shock, thermal resistance, and acid resistance were the tests conducted on fresh and hardened concrete for the mixes recorded above. Three specimens were casted for each test respectively for a mix. Geopolymer specimens were kept in open air in ambient room temperature (25–30 °C). Cement concrete specimens were dipped in water for curing until the day of testing. The tests on fresh and hardened concrete were conducted as per Indian codal provisions.

### 4.1. Slump Cone

Slump cone was conducted to find the workability of concrete. Both the geopolymer and cement fresh concretes with various types of fine aggregates were allowed to conduct the slump cone test. The test was conducted as per IS 1199-1959 [49].

### 4.2. Compression Strength and Split Tensile Strength

**Figure 1:**
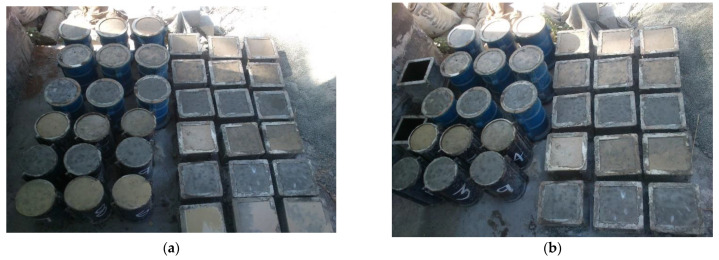
(**a**) GPC specimens, (**b**) CC specimens.

Cube and cylinder specimens of the respective mixes recorded in Table 4 and Table 5 were casted and tested. The images of casted specimens are shown in Figure 1a,b. The results obtained are discussed in clause 5.1. Testing was conducted on the casted specimen’s right at the 28th day of casting. Different embedded sand types along with coarse aggregate in geopolymer concrete and cement concrete failed at various loads. The variations of the load-carrying capacity for respective mixes were observed. The failure behavior of each specimen at different loads were observed and recorded. A strength test was conducted as per IS 516-1959 [50].

### 4.3. Durability Test

#### 4.3.1. Water Absorption Test

To find the percentage of water absorbed, 150 × 150 × 150 mm cube specimens of the mixes recorded in Table 4 and Table 5 were dipped in water for 48 h after the 28th day of casting. The weights of specimens in dry and wet states were recorded and the percentage of water absorbed by each specimen was calculated according to BS1881: part 5(8) [51].

#### 4.3.2. Acid Test

**Figure 2:**
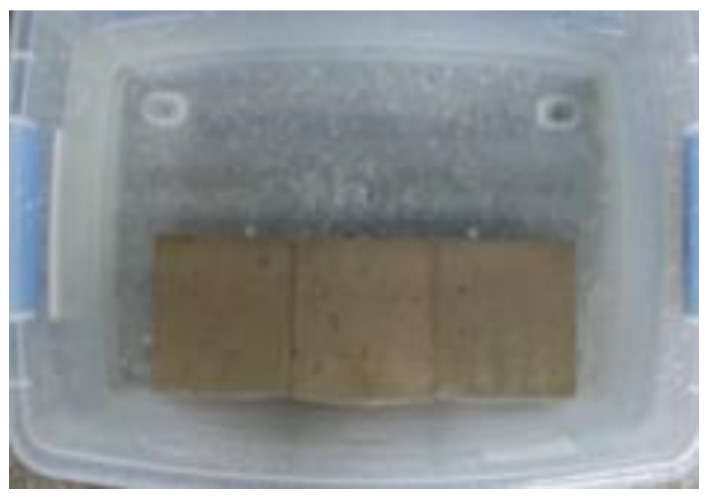
GPC specimens kept in sulfuric acid.

For the acid test (Figure 2), after demoulding, the specimens were weighed and dipped in a sulphuric acid (H_2_SO_4_) (Astra Chemicals Pvt. Ltd., Chennai, India) solution with a pH value of 0.2 for 25 days, as per the literature [52]. The specimens were taken out after 25 days of immersion in acid solution, washed in running water, and kept for 2 days in the atmosphere with constant weight. The weight loss and strength loss were monitored and recorded for three specimens of each mix. The recorded values were also compared with the control specimens that followed normal curing. The weight loss and residual compressive strength of concrete were used to measure its acid resistance [53].

#### 4.3.3. Water Permeability Test

**Figure 3:**
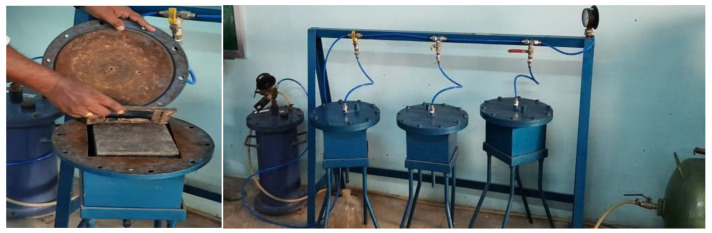
Water permeability apparatus and setup.

IS 3085:1965 [54] was referred to so as to conduct the water permeability test for the specimens cast for the drafted mixes. About 2 kg/cm^2^ of air pressure was applied for the constant flow of water for 100 h. The water permeability instrument setup is shown in Figure 3. The coefficient of permeability was calculated from the obtained results.

#### 4.3.4. Thermal Test

The compressive strength of geopolymer concrete and cement concrete mixes subjected to a temperature range of 200 °C and 400 °C for 24 h was observed and recorded. The cube specimens with a size of 150 × 150 × 150 mm were cast for the mentioned mixes after 28 days and kept in a heat oven at a temperature of 200 °C and 400 °C for 24 h. Beyond that, the amount of strength loss was determined by conducting compression tests on the specimens. The durability results are represented graphically in clause 5.2.

### 4.4. Cost Analysis

Even when a material performs well in all aspects, its cost must be reasonable to society to use it. Considering that, the costs of each mix were based on the South Indian market price. This would be most useful for persons who want to choose the materials based on the cost too. Clause 4.3 clearly discusses the costs incurred for respective mixes.

## 5. Results and Discussion

### 5.1. Workability and Strength

**Figure 4:**
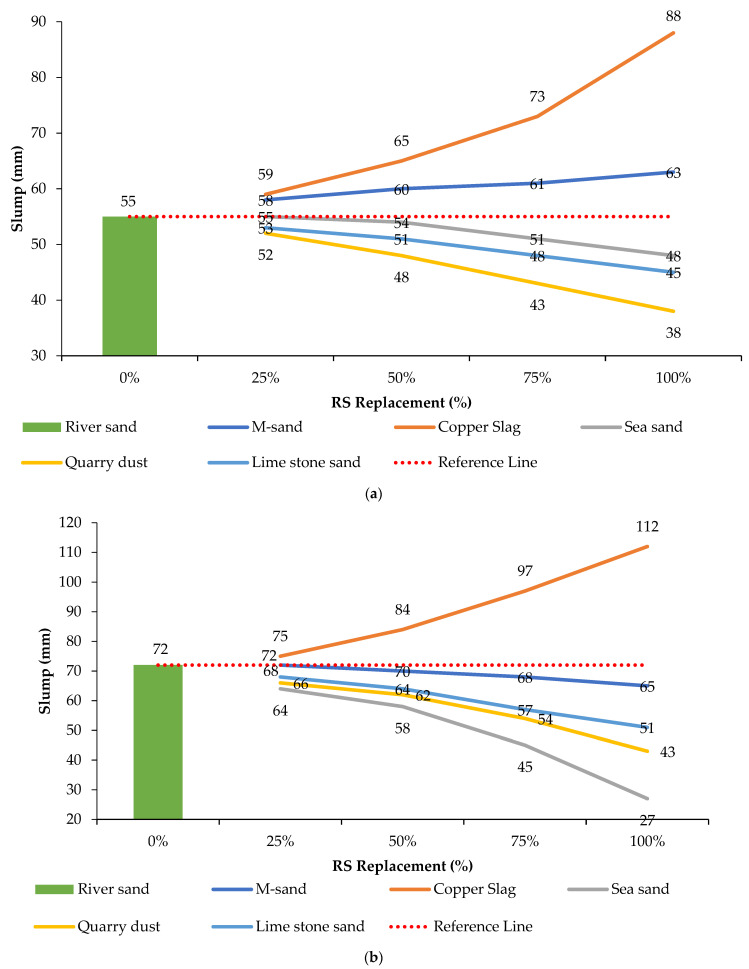
(**a**) The GPC—Slump value with respect to percentage of river sand replacement. (**b**) The CC—Slump value with respect to the percentage of river sand replacement.

Figure 4a,b shows the graphical representation of slump values for each of the GPC and CC mixes. M-sand and copper slag in GPC obtained good workability when compared to river sand mix. However, in CC mixes, copper slag alone gave higher workability when compared to river sand due to its glossy texture. M-sand in CC obtained a small fall of slump value. Sea sand, quarry dust, and limestone sand attained low workability in both GPC and CC due to its increase in water absorption capacity.

It can be observed that an increase in quarry dust, sea sand and limestone sand in GPC and CC mixes obtains a decrease in workability when compared to the control mix. To maintain workability levels similar to the control mix for respective mixes, extra water is needed. In the same way, water can be saved from highly workable concrete mixes with copper slag to maintain constant workability with the control mix. This study did not add or remove water from the designed proportion, so as to study the actual behavior of each fresh concrete mix.

**Figure 5:**
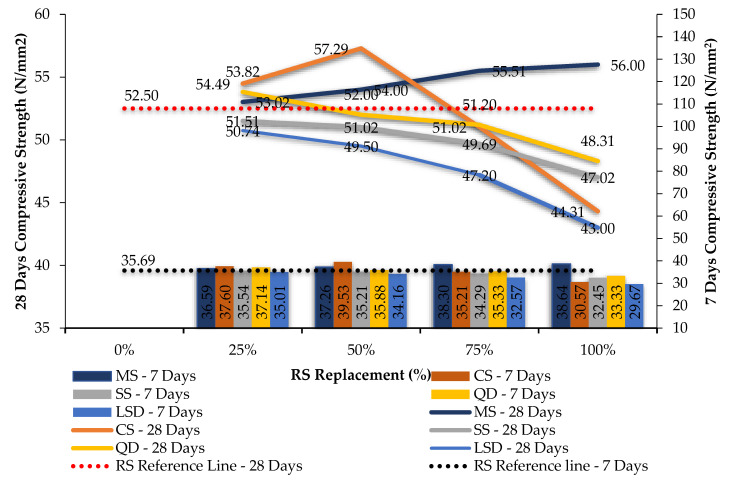
The 7- and 28-day compression test results for M_40_ GPC with various fine aggregates.

**Figure 6:**
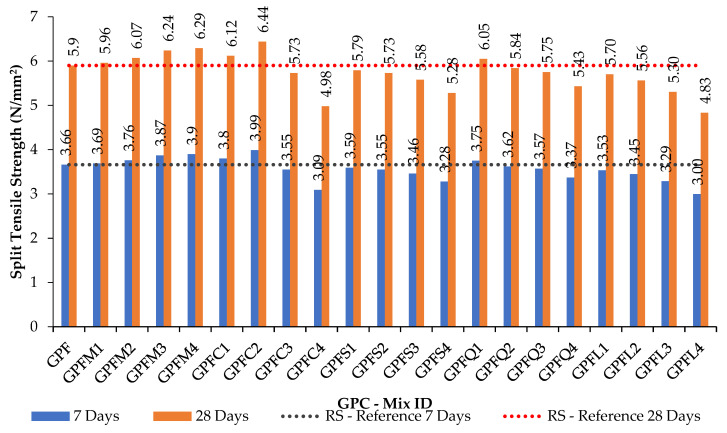
The 7- and 28-day split tensile test results for M_40_ GPC with various fine aggregates.

**Figure 7:**
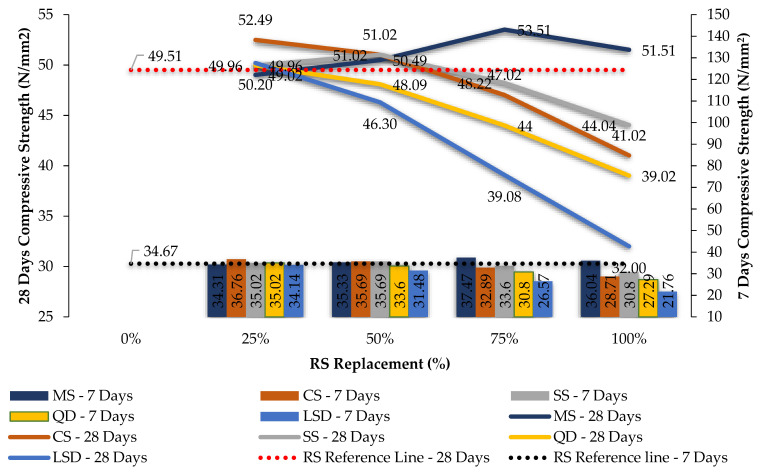
The 7- and 28-day compression test results for M_40_ CC with various fine aggregates.

**Figure 8:**
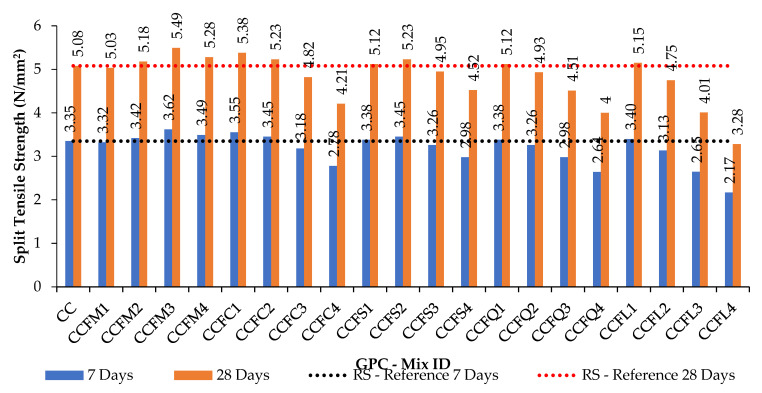
The 7- and 28-day split tensile test results for M_40_ CC with various fine aggregates.

The compression and split tension strength results of mixes shown in Table 4 and Table 5 are given in Table 6 and Table 7. Figure 5 and Figure 6 are the graphical representations of GPC’s strength achievement towards the compression and spit tensile loading. Figure 7 and Figure 8 are the graphs plotted to show the strength variation of all CC specimens. In all plotted graphs, the strength achieved by the control specimen was kept as a reference line. This clearly shows the increase or decrease of strength for each mix when compared to the control mix [15,16].

Considering the GPC test results, keeping the control specimen as a benchmark, M-sand at all replacement levels, copper slag at 50% replacement, and quarry dust at 25% obtained an increase in strength. Beyond 50% of copper slag and 25% of quarry dust, a decrease in strength was found at GPC. Limestone sand as the fine aggregate of GPC did not meet the strength levels of control specimens in all replacement levels. Considering the compressive strength, M-sand in the GPC mix got an increase in value when compared to the other sand types [55]. Significantly lesser strength was achieved for sea sand specimens [33]. The strength of mixes GPFM4, GPFC2, and GPFQ1 was considered to be optimized while using M-sand, copper slag, and quarry dust. These mixes achieved an increase in strength of about 6.67%, 9.12%, and 2.51% when compared to GPF. Even though copper slag achieved a strength greater than M-sand, due to its scarcity, M-sand can be advised to be used as a fine aggregate for GPC.

Considering the CC test results, comparing to the control mix, M-sand at 75% replacement, copper slag up to 25% replacement, sea sand up to 50% replacement, quarry dust at 25%, and limestone sand at 25% got an increase in strength. Beyond 75% of M-sand, 25% of copper slag, 50% of sea sand, and 25% of quarry dust, a decrease in strength was found in CC. Limestone sand as the fine aggregate of CC did not meet the strength of control specimens only at 25%, and in all replacement levels, it had a decrease in strength. The strength of mixes CCFM3, CCFC1, CCFS2, CCFQ1, and CCFL1 were considered to be optimized while using M-sand, copper slag, sea sand, quarry dust, and limestone sand. These mixes achieved an increase in strength of about 8.07%, 6.01%, 3.04%, 0.90%, and 1.40% when compared to CC, respectively.

Split tensile strength results for the GPC and CC mixes were found to get corresponding variations as found in compressive strength. Sea sand, copper slag, quarry dust, and limestone sand were unable to enhance strength more than the control mix in both GPC and CC in full replacement. Figure 5, Figure 6, Figure 7 and Figure 8 and Table 6 show the strength results of GPC and CC mixes with six types of fine aggregates. All the five types of sand materials are suited for CC but not GPC, except for M-sand, copper slag, and quarry dust.

### 5.2. Durability Test

The mixes that achieved higher compression values with different fine aggregates were selected to conduct durability tests. GPF, GPFM4, GPFC2, GPFS1, GPFQ1, and GPFL1 are the mixes considered in the side of GPC. Mixes CC, CCFM3, CCFC1, CCFS2, CCFQ1, and CCFL1 were considered in the CC side. The other mixes that did not get reasonable strength when compared with the control mixes were not subjected to durability tests.

### 5.3. Water Absorption

**Figure 9:**
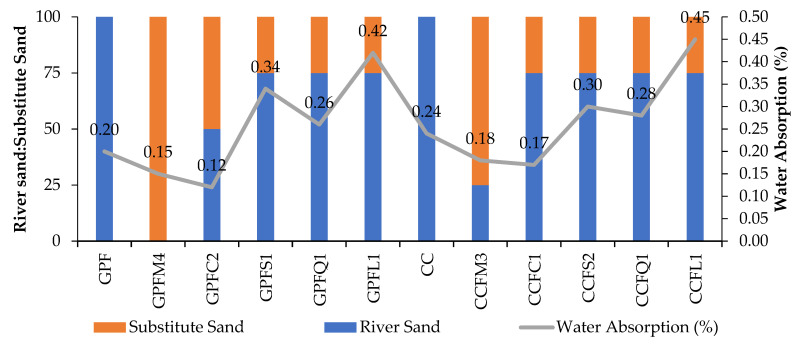
Percentage of water absorption in GPC and CC.

The strength properties of the mixes tested for geopolymer concrete and cement concrete are reflected in the durability test results. The results shown in Figure 9 include water absorption results. The absorption percentages of control mixes GPF50 and CC50 are 0.20% and 0.24%, respectively. The water absorption level of the GPC control specimen is 18.18% lower than the CC control specimen. It confirms the previous research statement that water absorption of CC is higher than GPC. The silica gel in GPC arrested the pores and prevented water absorption [56]. 

However, in loose aggregate concrete specimens, the absorption was found to be more. Both in geopolymer and cement concrete, M-sand and copper slag contained specimens which absorbed less water when compared to the control specimens. This clearly shows that M-sand and copper slag has naturally low water-absorbing capacities. Sea sand, quarry dust, and limestone sand absorbed a high percentage of water due to their absorption capacity. Figure 8 clearly shows the variation of water absorbed by GPC and CC with different fine aggregates.

### 5.4. Acid Attack

**Figure 10:**
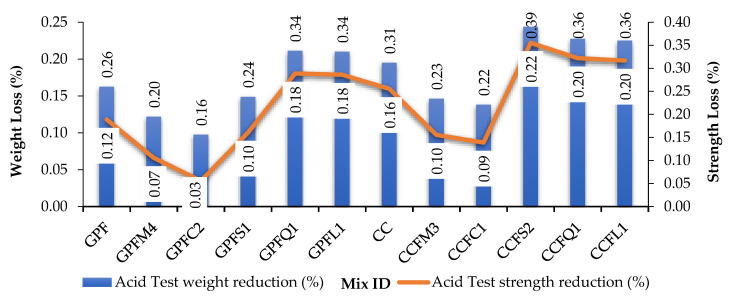
Acid test results of GPC and CC.

The surfaces of the specimens after taking out the acid solution are rough and do not resemble the control specimens. The specimens were weighed and tested under compression. The loss of strength and weight are noted in Table 7 and shown graphically in Figure 10. Earlier studies stated that GPC has better resistance towards acid attack [57,58]. The reduction of weight and strength of the GPC control specimen is 0.26% and 0.22%. Aluminosilicate-bonding in geopolymer concrete may be collapsed by the sulfuric acid attack; this may reduce the strength of GPC. Similarly, the reduction of weight and strength of the CC control specimen is 0.31% and 0.26%. 

In both GPC and CC, M-sand and copper slag-based specimens gave good results. Nearly 0.20% of weight loss of GPC and 0.23% of weight loss of CC was reduced by using M-sand, respectively. About 0.16% of weight loss was found in GPC and 0.22% of weight loss of CC was reduced by using copper slag, respectively. The weight loss using copper slag was too low, and may have happened due to some reaction between the copper slag and sulfate solution [42]. In GPC, M-sand and copper slag prevented 26% and 47% of weight loss. The same materials increased 29.60%, 34% resistance towards weight loss in CC. Similarly, M-sand and copper slag saved 25.60% and 51.42% of strength reduction in GPC and 26%, 31.10% resistance towards strength loss in CC. Sea sand in GPC and limestone sand in CC were met with huge losses in weight and strength [15,16,24,25,26,57,58,59].

### 5.5. Water Permeability

**Figure 11:**
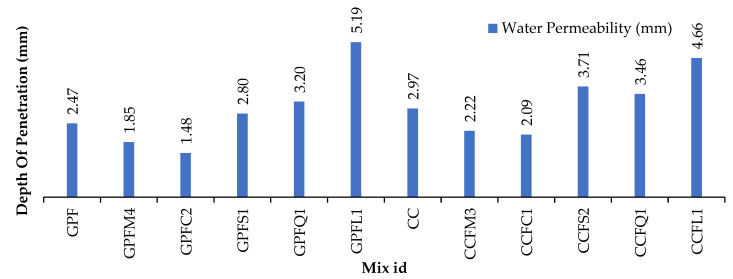
Co-efficient of permeability for GPC and cement concrete.

Water permeability under pressure was calculated and recorded in Table 7 and Figure 11. A clear increase and decrease of water penetration in specimens with varying fine aggregates of GPC and CC can be found in Figure 8. Specimens with good packing of materials resist water permeability. GPC specimens attained lower permeability values when compared to CC specimens. In both cases, M-sand and copper slag got dense packing and the remaining sand particles got an increase in water penetration due to their high water-absorbing capacity [41]. The permeability resistance of GPC and CC with sea sand, limestone sand and quarry dust is comparatively less. This resembles the water absorption test results. Quarry dust, although good at achieving strength, failed in water permeability.

### 5.6. Thermal Test

**Figure 12:**
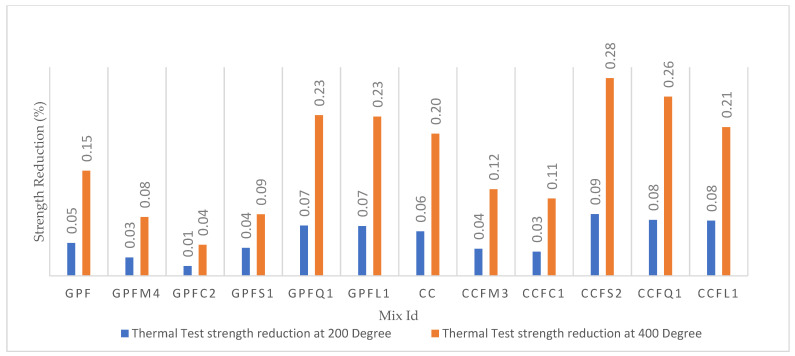
Thermal test results at 200 °C and 400 °C.

The thermal test results are shown in Table 7 and Figure 12. Both GPC and CC had a drop in strength beyond a temperature of 200 °C. This may have been due to the reactions within the concrete after 200 °C. About 0.20% to 0.50% of strength was reduced for the specimens under 400 °C. At 200 °C, the loss of strength for each specimen was comparatively less. Within 200 °C and 400 °C, the fall in strength was too high. The strength reduction in sea sand and limestone sand based on GPC is too high, and this may have been due to any reaction which happened within the concrete. M-sand and copper slag attained very low strength loss both in GPC and CC.

## 6. Cost Analysis

**Figure 13:**
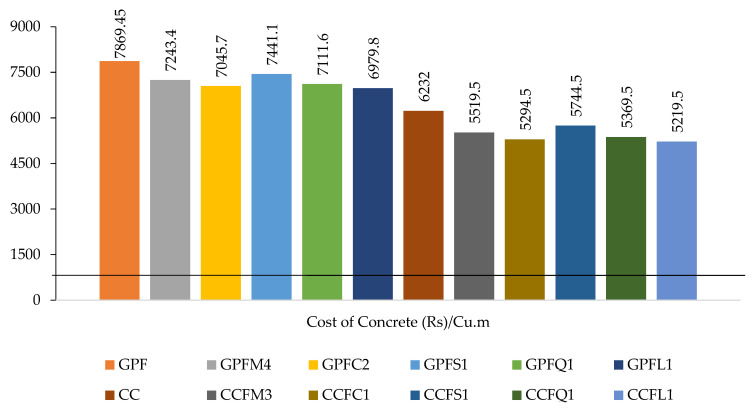
Cost analysis of GPC and cement concrete mixes.

The cost, strength, and durability of concrete are the three most significant aspects in construction. Civil engineering is always interested in any breakthrough that has a positive impact on these aspects. The variations in cost for the mixes discussed above are given in Table 8 and shown graphically in Figure 13. The cost for each mix was attained by considering the price of the material only on-site. For mineral admixtures, the price for one ton was considered. Additionally, for the other fine aggregates and coarse aggregates, the price for three tons was considered. These prices may vary in other parts of the world based on material availability. Prices for the GPC mixes for M_40_ grade concrete is nearly 23.22% higher than CC in cost. Although the cost is higher, ecofriendly concrete is GPC. Therefore, this cost variation will not have a huge impact on GPC mixes. Considering the rates aggregate-wise, M-sand was better than river sand in strength, and durability also had an effect with regard to cost. M-sand-based GPC and CC saved costs of about 8.30% and 14.86%, respectively. Mixes with M-sand obtained better results in strength and durability. Additionally, using it can result in a more low-cost concrete than river-sand-based concrete. The other types of fine aggregates also considerably reduce the cost of GPC and CC concretes.

## 7. Conclusions

The following conclusions can be taken from the outcomes reported in this study. Replacement of river sand in geopolymer concrete and cement concrete is possible using M-sand, copper slag, and quarry dust. The workability and strength of GPC- and CC-based concrete with M-sand and copper slag as fine aggregates were found to be good. Compared to copper slag, sea sand, quarry dust, limestone powder, and river sand, M-sand was found to be good in the workability and strength aspects. Up to 100% of the river sand could be replaced in GPC and up to 75% of river sand could be replaced in CC. Copper slag occupied a position next to M-sand. Nearly 50% of the copper slag can be replaced for GPC and 25% for CC. Quarry dust for both types of concrete is eligible only up to 25%, and the remaining goes for river sand. Sea sand in GPC improves the strength, but in durability studies, it failed to footprint, so it cannot be referred to as a good replacement material for river sand. Limestone sand was found to get a continuous drop in strength levels after 25%. Only the use of a small percentage of limestone sand below 25% is advised.

The depth of water absorption was reduced in GPC due to the gel-foam packing between the pores. Other than this, the fine aggregate materials, which are good in water absorption like sea sand, quarry dust, and limestone sand, were found to increase in water absorption value. This absorption of water may sometimes increase the mass of concrete and create internal damages in RCC structures. M-sand and copper slag-based GPC and CC were found to have low water absorption values.

Sulfuric acid attack in CC is aggressive. This type of acid attack may reduce the strength of concrete and also reduce its mass. Results clearly showed the domination of GPC in resisting sulfuric acid attack when compared to CC. A rough surface was found on the 28th day of CC specimens. Specimens of GPC did not deteriorate over its surface. The surface was found to be smooth and good after 28 days. The silica content in the GPC fills the pores and effectively resists the acid attack. The only substantial changes occurred in the strength of GPC specimens, and CC specimens exhibited larger changes. Similar to water permeability, river sand, M-sand, and copper slag-based GPC were found to be resistant towards sulfuric acid attack. The surface of the sea sand-based specimens was found to be rough when compared to other specimens, both in GPC and CC. Considerable losses in strength and weight were also found for limestone sand. 

For both the GPC and CC specimens, strength loss occurred within 200 °C to 400 °C. At 400 °C, the CC specimens were found to degrade slightly over their surface, and such an effect was not found in GPC specimens. The increase in temperature may have affected the chemical bonding of both GPC and CC specimens. The exact temperature at which the reaction occurs should be discovered in future. M-sand-based GPC and CC specimens performed well at both temperatures. Only a small percentage of strength loss was found.

M-sand as fine aggregate was found to be good in the aspects of strength and durability as well as cost-wise when compared to river sand. About 8.30% and 14.86% of the cost could be reduced in both GPC and CC specimens by using M-sand as a fine aggregate, which also gave an increase in strength. Even though copper slag, quarry dust, and sea sand had a significant effect on strength improvement, they did not shine in the durability aspects. Thus, M-sand was found to be a good alternative for river sand both in the GPC and CC structures.

## Figures and Tables

**Table 1:** Chemical properties of mineral admixtures.

Material	SiO_2_	Al_2_O_3_	Fe_x_O_y_	CaO	K_2_O	SO_3_	N_2_O	MgO	Loss of Ignition
Fly Ash	69.35	13.55	10.75	4.25	-	-	-	-	2.10
GGBS	34.43	13.58	0.42	42.60	0.3	3.67	-	5	-
OPC 53	22.24	4.10	4.45	64.40	-	2.52	0.51	1.20	0.58

**Table 2:** Physical properties of mineral admixtures.

Material	Fly Ash	GGBS	OPC 53
Specific Gravity	2.35	3.2	3.14
Specific Surface Area (m^2^/kg)	350	500	310

**Table 3:** Physical properties of aggregates.

Material	River Sand	M-Sand	Sea Sand	Quarry Dust	Copper Slag	Limestone Sand	Coarse Aggregate
Specific Gravity	2.65	2.6	2.62	2.65	3.2	2.55	2.70
Size (or) Gradation	II	II	III	IV	I	III	20 mm

**Table 4:** Mix proportions for M_40_ geopolymer concrete with various soil types.

Mix ID	Geopolymer Concrete Mixture Quantity (kg/m^3^)										
	CA	GGBS	FA	NaOH	Na_2_SiO_3_	RS	MS	CS	SS	QD	LSS
GPF	1158	208	208	57.5	172.5	757	0	0	0	0	0
GPFM1	1158	208	208	57.5	172.5	567.75	189.25	0	0	0	0
GPFM2	1158	208	208	57.5	172.5	378.5	378.5	0	0	0	0
GPFM3	1158	208	208	57.5	172.5	189.25	567.75	0	0	0	0
GPFM4	1158	208	208	57.5	172.5	0	757	0	0	0	0
GPFC1	1158	208	208	57.5	172.5	567.75	0	189.25	0	0	0
GPFC2	1158	208	208	57.5	172.5	378.5	0	378.5	0	0	0
GPFC3	1158	208	208	57.5	172.5	189.25	0	567.75	0	0	0
GPFC4	1158	208	208	57.5	172.5	0	0	757	0	0	0
GPFS1	1158	208	208	57.5	172.5	567.75	0	0	189.25	0	0
GPFS2	1158	208	208	57.5	172.5	378.5	0	0	378.5	0	0
GPFS3	1158	208	208	57.5	172.5	189.25	0	0	567.75	0	0
GPFS4	1158	208	208	57.5	172.5	0	0	0	757	0	0
GPFQ1	1158	208	208	57.5	172.5	567.75	0	0	0	189.25	0
GPFQ2	1158	208	208	57.5	172.5	378.5	0	0	0	378.5	0
GPFQ3	1158	208	208	57.5	172.5	189.25	0	0	0	567.75	0
GPFQ4	1158	208	208	57.5	172.5	0	0	0	0	757	0
GPFL1	1158	208	208	57.5	172.5	567.75	0	0	0	0	189.25
GPFL2	1158	208	208	57.5	172.5	378.5	0	0	0	0	378.5
GPFL3	1158	208	208	57.5	172.5	189.25	0	0	0	0	567.75
GPFL4	1158	208	208	57.5	172.5	0	0	0	0	0	757

**Table 5:** Mix Proportion for M40 cements concrete with various soil types.

Mix ID	Cement Concrete Mixture Quantity (kg/m^3^)								
	CA	Cement	Water	RS	MS	CS	SS	QD	LSS
CC	1081	450	190	678.68	0	0	0	0	0
CCFM1	1081	450	190	509.01	169.67	0	0	0	0
CCFM2	1081	450	190	339.34	339.34	0	0	0	0
CCFM3	1081	450	190	169.67	509.01	0	0	0	0
CCFM4	1081	450	190	0	678.68	0	0	0	0
CCFC1	1081	450	190	509.01	0	169.67	0	0	0
CCFC2	1081	450	190	339.34	0	339.34	0	0	0
CCFC3	1081	450	190	169.67	0	509.01	0	0	0
CCFC4	1081	450	190	0	0	678.68	0	0	0
CCFS1	1081	450	190	509.01	0	0	169.67	0	0
CCFS2	1081	450	190	339.34	0	0	339.34	0	0
CCFS3	1081	450	190	169.67	0	0	509.01	0	0
CCFS4	1081	450	190	0	0	0	678.68	0	0
CCFQ1	1081	450	190	509.01	0	0	0	169.67	0
CCFQ2	1081	450	190	339.34	0	0	0	339.34	0
CCFQ3	1081	450	190	169.67	0	0	0	509.01	0
CCFQ4	1081	450	190	0	0	0	0	678.68	0
CCFL1	1081	450	190	509.01	0	0	0	0	169.67
CCFL2	1081	450	190	339.34	0	0	0	0	339.34
CCFL3	1081	450	190	169.67	0	0	0	0	509.01
CCFL4	1081	450	190	0	0	0	0	0	678.68

Annotations: CA—Coarse Aggregate; GGBS—Ground Granulated Blast Slag; FA—Fly ash; RS-River Sand; MS—M-Sand; CS—Copper Slag; SS—Sea Sand; RM—Quarry Dust; LSS—Limestone sand.

**Table 6:** The 28-day test results for GPC and CC with various fine aggregates.

Mix ID	Geopolymer Concrete					Mix ID	Cement Concrete				
	Slump (mm)	Compressive Strength (kN/mm^2^)		Split Tensile Strength (kN/mm^2^)			Slump (mm)	Compressive Strength (kN/mm^2^)		Split Tensile Strength (kN/mm^2^)	
		7 Days	28 Days	7 Days	28 Days			7 Days	28 Days	7 Days	28 Days
GPF	55	36.23	52.50	3.66	5.9	CC	72	34.67	49.51	3.35	5.08
GPFM1	58	36.59	53.02	3.69	5.96	CCFM1	72	34.31	49.02	3.32	5.03
GPFM2	60	37.26	54.00	3.76	6.07	CCFM2	70	35.33	50.49	3.42	5.18
GPFM3	61	38.30	55.51	3.87	6.24	CCFM3	68	37.47	53.51	3.62	5.49
GPFM4	63	38.64	56.00	3.9	6.29	CCFM4	65	36.04	51.51	3.49	5.28
GPFC1	59	37.60	54.49	3.8	6.12	CCFC1	75	36.76	52.49	3.55	5.38
GPFC2	65	39.53	57.29	3.99	6.44	CCFC2	84	35.69	51.02	3.45	5.23
GPFC3	73	35.21	51.02	3.55	5.73	CCFC3	97	32.89	47.02	3.18	4.82
GPFC4	88	30.57	44.31	3.09	4.98	CCFC4	112	28.71	41.02	2.78	4.21
GPFS1	55	35.54	51.51	3.59	5.79	CCFS1	64	35.02	49.96	3.38	5.12
GPFS2	54	35.21	51.02	3.55	5.73	CCFS2	58	35.69	51.02	3.45	5.23
GPFS3	51	34.29	49.69	3.46	5.58	CCFS3	45	33.60	48.22	3.26	4.95
GPFS4	48	32.45	47.02	3.28	5.28	CCFS4	27	30.80	44.04	2.98	4.52
GPFQ1	52	37.14	53.82	3.75	6.05	CCFQ1	66	35.02	49.96	3.38	5.12
GPFQ2	48	35.88	52.00	3.62	5.84	CCFQ2	62	33.60	48.09	3.26	4.93
GPFQ3	43	35.33	51.20	3.57	5.75	CCFQ3	54	30.80	44.00	2.98	4.51
GPFQ4	38	33.33	48.31	3.37	5.43	CCFQ4	43	27.29	39.02	2.64	4.00
GPFL1	53	35.01	50.74	3.53	5.70	CCFL1	68	34.14	50.20	3.40	5.15
GPFL2	51	34.16	49.50	3.45	5.56	CCFL2	64	31.48	46.30	3.13	4.75
GPFL3	48	32.57	47.20	3.29	5.30	CCFL3	57	26.57	39.08	2.65	4.01
GPFL4	45	29.67	43.00	3.00	4.83	CCFL4	51	21.76	32.00	2.17	3.28

**Table 7:** Durability test results for geopolymer concrete and cement concrete mixes.

Mix ID	Water Absorption (%)	Acid Test		Water Permeability (mm)	Thermal Test	
		Weight Reduction (%)	Strength Reduction (%)		Strength Reduction at 200 °C (%)	Strength Reduction at 400 °C (%)
GPF	0.20	0.26	0.22	2.47	0.09	0.28
GPFM4	0.15	0.20	0.17	1.85	0.07	0.21
GPFC2	0.12	0.16	0.13	1.48	0.05	0.17
GPFS1	0.64	0.45	0.70	5.27	0.28	0.62
GPFQ1	0.18	0.23	0.20	2.21	0.08	0.25
GPFL1	0.42	0.34	0.46	5.19	0.18	0.59
CC	0.24	0.31	0.26	2.97	0.11	0.34
CCFM3	0.18	0.23	0.20	2.22	0.08	0.25
CCFC1	0.17	0.22	0.19	2.09	0.07	0.24
CCFS2	0.30	0.39	0.33	3.71	0.13	0.42
CCFQ1	0.23	0.30	0.25	2.84	0.10	0.32
CCFL1	0.45	0.36	0.50	4.66	0.20	0.53

**Table 8:** Cost analysis for geopolymer concrete and cement concrete mixes.

Mix ID	GPF	GPFM4	GPFC2	GPFS1	GPFQ1	GPFL1
Cost of Concrete (Rs)/m^3^	7869.45	7243.40	7045.70	7441.1	7111.60	6979.80
**Mix ID**	**CC**	**CCFM3**	**CCFC1**	**CCFS1**	**CCFQ1**	**CCFL1**
Cost of Concrete (Rs)/m^3^	6232	5369.50	5294.50	5744.50	5519.50	5219.50

## Data Availability

Data sharing not applicable.
